# Hierarchical Multi-Scale Feature Fusion Network with Implicit Neural Representation and Mamba for Cross-Modality MRI Synthesis

**DOI:** 10.3390/s26061901

**Published:** 2026-03-18

**Authors:** Zhihao Luo, Jun Lyu

**Affiliations:** School of Computer and Control Engineering, Yantai University, Yantai 264005, China; fahaxiki@s.ytu.edu.cn

**Keywords:** magnetic resonance imaging (MRI), medical image synthesis, Mamba, implicit neural representation (INR)

## Abstract

Magnetic resonance imaging (MRI), a widely adopted modality in clinical practice, enables the acquisition of multi-contrast images from the same anatomical structure, commonly referred to as multimodal images. Integrating these diverse modalities is crucial for enhancing model performance across a variety of medical image analysis tasks. However, in real-world clinical scenarios, it is often impractical to acquire all MRI modalities simultaneously due to factors such as patient discomfort, time constraints, and scanning costs. As a result, synthesizing missing modalities from available ones has emerged as an effective solution. To address these challenges, we propose HMF-MambaINR, a hierarchical multi-scale feature fusion network for cross-modality MRI synthesis. The model integrates Mamba-based Selective State Space Modeling (SSM) and implicit neural representation (INR) to capture long-range dependencies and enable continuous spatial reconstruction. A Multi-Feature Extraction Block (MFEB) captures local and global representations via multi-scale receptive fields, while a Modulation Fusion Module (MFM) adaptively fuses multi-modal features with dynamic weighting. Extensive experiments show that HMF-MambaINR surpasses state-of-the-art CNN-, Transformer-, and Mamba-based methods in synthesizing missing MRI modalities. Notably, the synthesized MRI images received positive feedback from radiologists in terms of image quality, contrast, and structural contour accuracy, highlighting the potential of the proposed method as a practical tool for clinical applications.

## 1. Introduction

Magnetic resonance imaging (MRI) has become an indispensable tool in clinical disease diagnosis, offering high-resolution structural and functional information without the risks associated with ionizing radiation. In clinical practice, multiple MRI modalities are typically acquired by configuring task-specific scanning parameters, such as T1-weighted (T1), contrast-enhanced T1-weighted (T1c), T2-weighted (T2), and T2 fluid-attenuated inversion recovery (FLAIR) images. Owing to the distinctive soft tissue contrast and feature representation of different modalities, which allow for the reflection of tissue anatomy and pathological changes from multiple perspectives, integrating the supplementary data obtained from different MRI sequences facilitates more effective medical image analysis [1,2]. However, in practical clinical settings, obtaining all required imaging modalities is frequently hindered by variations in scanning protocols, patient discomfort, and the substantial costs associated with imaging procedures. Therefore, the problem of cross-modality MRI image synthesis for missing modalities has attracted considerable interest across clinical and academic domains in recent years.

Cross-modality medical image synthesis aims to generate missing target modalities by learning a mapping from the available source modality to the desired one. Owing to the high dimensionality of medical images, their complex anatomical structures, and the nonlinear contrast variations across modalities, cross-modality synthesis becomes particularly challenging when the target modality is unavailable during inference [3]. In recent times, deep learning has grown to become the prevailing approach for medical image synthesis, largely due to its powerful capacity for approximating complex nonlinear functions. By performing nonlinear transformations in intermediate feature spaces, neural networks learn to map source modality images to their corresponding target modalities [4,5], effectively addressing many of the challenges inherent in this task. Early works primarily leveraged convolutional neural networks (CNNs), which apply local, spatially invariant filters to extract features, demonstrating superior representational power over traditional methods [6]. Subsequent advancements introduced generative adversarial networks (GANs), which incorporate adversarial loss to better model complex anatomical structures, leading to further performance improvements [7]. However, convolutional architectures inherently possess limited receptive fields, restricting their capacity to capture long-range dependencies and global contextual relationships [8]. To overcome these challenges, recent methods have adopted Transformer-based architectures that utilize self-attention mechanisms to dynamically model global context by computing attention weights among all token pairs [9]. This approach significantly improves the network’s capacity to capture long-range dependencies, enhancing performance on high-level tasks such as medical image synthesis [10]. Nevertheless, the self-attention mechanism incurs quadratic complexity with respect to the input size, which hinders the scalability of Transformers to high-resolution image data. Recently, the emergence of the improved selective structured state space model (Mamba) [11] provides a new solution to the above problem. Mamba achieves selective attention to key positions while maintaining linear computational complexity, combining local modeling efficiency with global modeling capabilities. This architecture decreases computational demands and boosts inference efficiency.

Current research efforts have started investigating the implementation of Mamba in multi-modal image fusion tasks [12,13]. Atli et al. [14] proposed a Mamba-based medical image synthesis model that integrates hybrid-channel Mamba blocks with CNNs, effectively capturing both short-range and long-range contextual information in medical images. However, the results generated by existing methods still suffer from issues such as blurred local textures and insufficient detail restoration. The reason is that existing methods fail to fully leverage multi-scale semantic information in multimodal medical images, which hinders the model’s capacity to accurately represent critical structures. Indeed, multi-scale feature fusion has been widely adopted in various vision tasks to enhance both contextual understanding and fine-grained detail modeling. Examples include multi-scale context modules with atrous mechanisms for RGB-T salient object detection [15], hierarchical multi-scale learning in brain tumor segmentation [16], and dynamic multi-scale fusion for improved medical image assessment [17]. In addition, existing Mamba-based synthesis models typically rely on discrete pixel-level or voxel-level representations, which inherently limit their ability to recover fine-grained details [18]. Given the recent excellent performance of implicit neural representation (INR) in encoding images into continuous functional representations [19,20,21], we propose to combine Mamba’s global sequence modeling capabilities with INR’s high-fidelity reconstruction capabilities in continuous space, and further introduce a multi-stage, multi-scale feature fusion mechanism to gradually guide the image synthesis process from coarse to fine. Unlike hybrid Transformer-CNN architectures that operate on fixed discrete grids, the combination of Mamba and implicit neural representations offers complementary advantages. Mamba excels at efficient global sequence modeling and long-range dependency capture, while implicit neural representations encode images as continuous functions, enabling more accurate reconstruction of fine structures and boundaries. This synergy forms a more effective representation framework and directly addresses the limitations of discrete pixel-level representations in current Mamba-based synthesis models. This design effectively solves the problem that existing methods are difficult to balance between structure and detail modeling, and provides a novel and promising solution for synthesizing multimodal medical images, which can be used not only to augment datasets for research but also to support downstream clinical tasks such as segmentation, registration, and diagnostic decision-making.

Building on these foundations, we propose a hierarchical multi-scale feature fusion network with Mamba and implicit neural representation (HMF-MambaINR). HMF-MambaINR selectively inserts the residual hybrid mamba block(RHMB) module to capture spatial contextual information and employs channel mixing layers for deep feature interaction across channels. Subsequently, a Multi-Feature Extraction Block (MFEB) is introduced to capture both fine-grained local textures and high-level global semantics by integrating multi-scale contextual representations across different feature hierarchies, and leverage the Modulation Fusion Module (MFM) module fine-grained integration of features across multiple scales, facilitating the fusion of spatially diverse structural and contextual information. Finally, an implicit neural representation block (INRB) maps the fused features into a continuous image generation function, enabling the synthesis of medical images with enhanced structural detail and rich tissue textures.

The primary contributions can be summarized as follows:

1. We propose a novel cross-modality MRI synthesis model (HMF-MambaINR) based on Mamba and INR to synthesize missing target domain images.
2. We design an innovative MFEB, which captures multi-scale features through multi-scale receptive fields and adaptively adjusts the weights of features at each scale based on their content, learning and understanding the complementary information between multiple features.
3. We introduce the MFM, which effectively facilitates multi-scale feature integration, alleviates feature sparsity issues encountered during the decoding stages, and thereby enhances the overall network performance.
4. We incorporate INR to model the continuous mapping between multi-modal feature and the image space, significantly enhancing the model’s representational capacity and its ability to synthesize fine-grained anatomical structures.

## 2. Related Works

### 2.1. Medical Image Synthesis

The objective of cross-modality medical image synthesis is to transform source modality images into corresponding missing target modalities. Several approaches have been introduced recently to overcome this problem. For example, Nie et al. [22] trained a fully convolutional network using an adversarial learning strategy to predict CT images from corresponding MR images. Similarly, Wei et al. [23] proposed a three-dimensional fully convolutional architecture was introduced for FLAIR image synthesis by learning mappings from other available MR modalities. Dar et al. [6] presented an approach powered by a conditional GAN architecture, utilizing pixel-level and cyclic consistency loss functions for multi-modal MRI image synthesis. However, these approaches predominantly concentrate on synthesizing medical images from a single source modality. To enhance synthesis performance using multiple modalities, numerous techniques for synthesizing images across multiple modalities have emerged. Zhou et al. [24] designed modality-specific networks to capture features of individual modalities and employed a layer-wise fusion strategy, further boosting the model’s effectiveness in multimodal image synthesis. Peng et al. [25] proposed a reliability-aware integration and inter-modal enhancement framework, which effectively utilized synergistic features across distinct data sources to generate high-quality target modality images. Li et al. [26] further incorporated boundary-sensitive pretraining combined with multi-scale fine-tuning strategies, enabling their model to generalize across both paired and unpaired MRI synthesis tasks.

### 2.2. Transformers and SSM Models in Medical Imaging

In recent years, Transformer-based approaches have gained significant traction in computer vision tasks [9], demonstrating strong potential in medical image segmentation, reconstruction, and synthesis. Dalmaz et al. [10], who integrated adversarial training with a Transformer-based generator for cross-modality synthesis; Yan et al. [27], who proposed a Swin Transformer architecture to alleviate boundary artifacts from small patch sizes; Liu et al. [28] developed a hierarchical, cross-contrast attention framework that models contrastive relationships for high-quality synthesis, while Zhang et al. [29] extracted modality-invariant and modality-specific representations to generate anatomically consistent images. Despite these advances in context modeling and image quality, the high computational cost of global interactions limits their clinical application.

As a promising alternative, selective state space models (SSMs) offer long-range dependency capture with low complexity. Recent applications in medical imaging include segmentation [30,31], classification [32], and synthesis [14]. For instance, Ma et al. [33] introduced a CNN-SSM hybrid for robust heterogeneous image processing, Yue et al. [32] proposed a Conv-SSM dual-branch module for multi-level feature extraction and improved classification, and Xing et al. [34] developed a tri-directional Mamba module for sequential and multi-scale modeling in 3D volumes. Atli et al. [14] pioneered SSM for modality translation, enabling efficient cross-modal synthesis. However, further enhancements can be achieved by integrating multi-stage and multi-scale features most relevant to target modality synthesis.

### 2.3. Implicit Neural Representation

INR is an emerging coordinate-based technique that leverages multilayer perceptrons (MLPs) to model continuous-domain signals. Unlike traditional methods that explicitly store signal values at each pixel or voxel, INR learns a continuous mapping from spatial coordinates to signal intensities, thereby achieving a compact and implicit encoding of visual data [35]. Originally developed for 3D vision applications, INR has recently garnered increasing attention in 2D image tasks. Li et al. [36] introduced a reference-aware attention module based on INR to achieve super-resolution of any scale. Gu et al. [37] introduced a self-distillation-based INR method that amplifies capillary details to facilitate retinal vessel segmentation for ophthalmic disease diagnosis. Wei et al. [38] combined uncertainty-guided sampling with INR to learn a mapping from feature–coordinate pairs to segmentation outputs. Recently, Feng et al. [39] first attempted to establish a continuous mapping of synthetic aperture radar (SAR) features to optical images through INR. However, their method is built upon CNN architectures, which are limited by the local receptive field and limited modeling capabilities of convolution and are difficult for capturing global contextual relationships and complex cross-modal nonlinear mapping relationships.

## 3. Methodology

### 3.1. Overview

**Figure 1:**
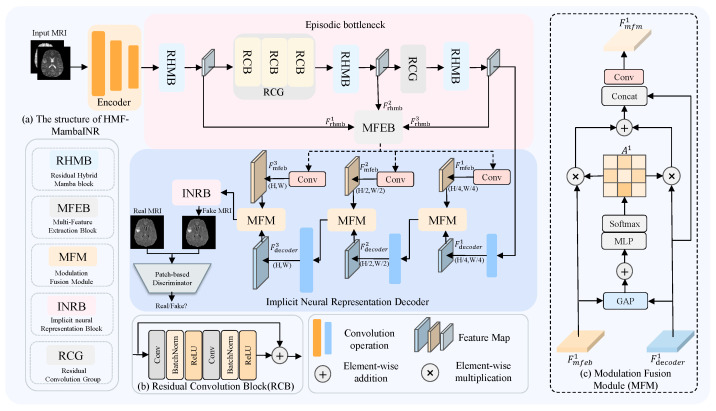
(**a**) Network architecture of HMF-MambaINR. The proposed model consists of an encoder, an episodic bottleneck, and a decoder module to synthesize images of the target modality generated from source-modality data. The encoder extracts features, while the Episodic bottleneck employs RHMB and RCG to capture contextual information, and the MFEB learns and understands the complementary information between multiple features. The decoder module leverages the MFM to facilitate multi-scale feature aggregation, and employs INRB to continuously map the fused features to the image domain. (**b**) Architecture of the RCB. (**c**) Architecture of the MFM.

As illustrated in Figure 1a, the proposed HMF-MambaINR architecture consists of an encoder, an episodic bottleneck, and a decoder, which together synthesize target-modality images from input source-modality images. The encoder comprises three stages, each based on a standard CNN block, consisting of a convolutional layer, batch normalization, and a ReLU activation function. The bottleneck module is primarily composed of three subcomponents: RHMB, CRG, and MFEB. The RHMB intermittently inserted at three critical positions: the beginning, middle, and end, to enhance the modeling of deep semantic features. The key features extracted from the three RHMB stages are fed into the MFEB, which is used to extract complementary information of features at different stages. Residual convolution group(RCG) uses a standard residual CNN module as its basic structure. The decoder module mainly consists of MFM and INRB. MFM is used to dynamically fuse features of different scales and enhance the expression of key features. INRB module uses continuous space mapping mechanism for image synthesis. The rest of the module retains the same CNN block structure as the encoder. The detailed architectural designs and implementation specifics of each key module are further elaborated in the subsequent sections.

### 3.2. Residual Hybrid Mamba Block

**Figure 2:**
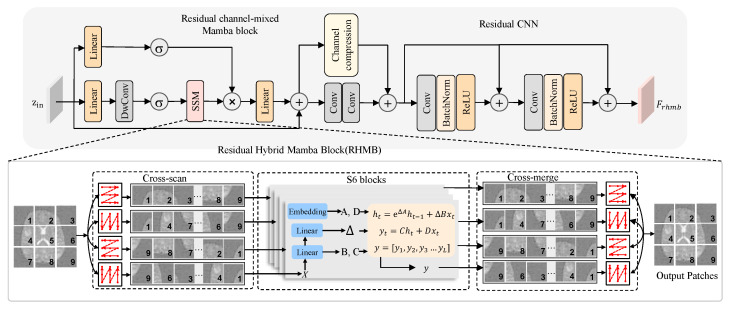
The detailed architecture of the RHMB. Numbers indicate different scanning directions.

The RHMB effectively exploits the sensitivity of SSM to long-range contextual dependencies, while leveraging the local accuracy advantages of CNN, significantly enhancing overall performance without introducing additional computational overhead. As shown in Figure 2, the RHMB consists of three key components: First, the SSM layer for capturing long-range contextual information; second, the channel mixing layer for modeling dependencies between feature map channels; and third, the convolutional layer for extracting local features. In the RHMB block, the input feature map $f\in R^{W^{\prime }\times H^{\prime }\times C}$ is first tokenized into $P=\frac{H^{\prime }W^{\prime }}{p^{2}}$ disjoint blocks with dimensions (p,p), resulting in a sequence $z_{\mathrm{in}}$. In the first branch, the sequence is linearly embedded for the calculation of the gating variable *G*:

(1) $$G=\sigma (\mathrm{Lin}\left(z_{\mathrm{in}}\right))$$

where σ denotes an activation function. In the second branch, the input sequence is first linearly embedded, then undergoes feature mixing via depthwise separable convolution, and finally, it is projected through the SSM layer:

(2) $$M=\mathrm{SSM}(\sigma \left(\mathrm{DWConv}\left(\mathrm{Lin}\left(z_{\mathrm{in}}\right)\right)\right))$$

The SSM layer in the equation is built upon the selective state space sequence model. Specifically, the sequence of patches is first extended by performing selective scanning in multiple directions of the 2D feature map. Then, the resulting sequence is processed using state space modeling, and finally, the processed sequence is merged back. In this process, we construct a discretized state space model:

(3) h[n]=Ah[n−1]+Bz[n]

(4) $$\bar{z}\left[n\right]=Ch\left[n\right]+Dz\left[n\right]$$

In this context, *h* denotes the hidden state, *z* represents the source sequence, and $\bar{z}$ refers to the target sequence, where *n* stands for the sequence index, while $A\in R^{N\times N}$, $B\in R^{N\times 1}$, $C\in R^{1\times N}$, and D∈R are the learnable SSM parameters. *N* is the dimensionality of the state representation.

After applying the Hadamard product for gating *M*, a linear projection is performed, followed by a residual addition with the input sequence:

(5) $$z_{\mathrm{SSM}}=z_{\mathrm{in}}+\mathrm{Lin}\left(G⊙M\right)$$

To further capture the context between different feature channels, $z_{\mathrm{SSM}}$ will pass through the channel mixing layer:

(6) $$z_{\mathrm{cm}}=\mathrm{cmConv}\left(z_{\mathrm{SSM}}\right)+\mathrm{Conv}\left(z_{\mathrm{SSM}}\right)$$

The channel mixing layer is implemented using a 1×1 convolution. Finally, a residual CNN block takes the processed features and generates the output:

(7) $$F_{rhmb}=z_{\mathrm{cm}}+RCNN\left(z_{\mathrm{cm}}\right)$$

The RCNN module is composed of two repeated CNN blocks, each containing a convolutional layer followed by batch normalization and a ReLU activation.

### 3.3. Multi-Feature Extraction Block

**Figure 3:**
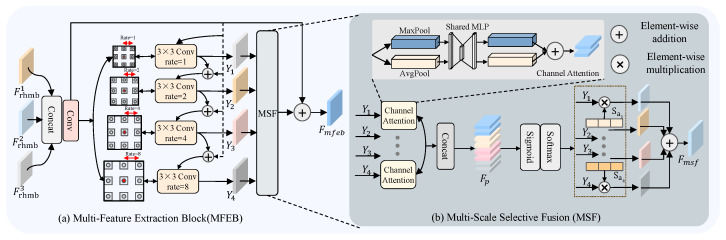
The comprehensive layout of the MFEB and MSF. Arrows indicate the direction of feature propagation.

As illustrated in Figure 3a, we introduce a Multi-Feature Extraction Block (MFEB) designed to capture multi-scale features more effectively and enhance the model’s capacity to represent both semantic and structural information. This structure fully leverages feature information from different stages while capturing multi-scale feature representations, enabling the fusion of shallow local textures and deep semantic features, consequently strengthening the model’s competence for multi-level understanding and representation of image content.

First, we concatenate the feature outputs from the three stages of the RHMB module to serve as the input for subsequent multi-scale extraction process. The features from the three stages are specifically represented as $F_{rhmb}^{1}\in R^{W^{\prime }\times H^{\prime }\times C}$, $F_{rhmb}^{2}\in R^{W^{\prime }\times H^{\prime }\times C}$, and $F_{rhmb}^{3}\in R^{W^{\prime }\times H^{\prime }\times C}$. These features are stacked along the channel dimension, and the resulting concatenated feature map is then passed through a 1×1 convolution operation for dimensionality reduction, producing an intermediate feature map $F_{\mathrm{tamp}}\in R^{W^{\prime }\times H^{\prime }\times C}$.

(8) $$F_{\mathrm{tamp}}=\mathrm{Conv}\left(\mathrm{concat}\left(F_{rhmb}^{1},F_{rhmb}^{2},F_{rhmb}^{3}\right)\right)$$

The input feature map $F_{\mathrm{tamp}}$ undergoes a series of four 3×3 dilated convolutions with distinct dilation factors to extract features across multiple scales $Y_{i}$·$Y_{i}\in R^{W^{\prime }\times H^{\prime }\times C}$.

(9) $$Y_{i}=\left\{\begin{array}{cc} {\mathrm{Conv}}_{3\times 3,\mathrm{rate}=1}\left(F_{\mathrm{tamp}}\right) & \mathrm{if}\;i=1,\\ {\mathrm{Conv}}_{3\times 3,\mathrm{rate}=2^{\left(i-1\right)}}\left(F_{\mathrm{tamp}}+Y_{i-1}\right) & \mathrm{if}\;1<i\leq 4. \end{array}\right.$$

In this design, *i* denotes the number of dilated convolutions, which is set to 4 based on experimental configuration. This design preserves the network’s depth while expanding its width, thereby enabling the extraction of fine-grained structural details as well as abstract contextual representations. Following feature extraction, we employ a Multi-scale selective fusion (MSF) module to integrate the multi-scale features $Y_{i}$. The details of MSF are shown in Figure 3b. First, global average pooling and global max pooling are performed on the four features from the previous stage to obtain their respective average channel weights. The results of pooling are represented as $P_{i}^{A}\in R^{C\times 1}$ and $P_{i}^{M}\in R^{C\times 1}$. Then, a convolution operation is applied, and the outputs from max pooling and average pooling are individually summed:

(10) $$P_{a_{i}}={\mathrm{Conv}}_{i}\left(P_{i}^{A}\right)+{\mathrm{Conv}}_{i}\left(P_{i}^{M}\right)\;1\leq i\leq 4$$

Here, $P_{a_{i}}$ represents the fused feature of the i-th multi-scale feature extracted in the preceding stage, and the index i∈{1,2,3,4} corresponds to the four multi-scale features. Than, the results are concatenated into $F_{p}=\left[P_{a_{1}},P_{a_{2}},P_{a_{3}},P_{a_{4}}\right]\in R^{4C\times 1}$. The weights are then mapped to the range of 0 to 1 using the sigmoid activation function, followed by a softmax operation applied to normalize the values at the same position across the multi-scale average channel weights, thereby achieving consistency modeling across scales:

(11) $$S_{a_{i}}^{j}=\frac{e^{P_{a_{i}}^{j}}}{e^{P_{a_{1}}^{j}}+e^{P_{a_{2}}^{j}}+e^{P_{a_{3}}^{j}}+e^{P_{a_{4}}^{j}}}\;1\leq i\leq 4$$

Here, $S_{a_{i}}^{j}$ represents the *j*-th element within the channel attention map, with the sum of all elements equal to 1. $P_{a_{i}}^{j}$ denotes the *j*-th element of $P_{a_{i}}$. Subsequently, the features are weighted by their corresponding normalized coefficients and then summed to produce the updated multi-scale features:

(12) $$F_{msf}=\sum_{i=(1,2,3,4)}S_{a_{i}}^{j}\cdot Y_{i}$$

where $F_{mfeb}$ denotes the fused feature map. Finally, the processed features are added to the initial features, and the resulting sum is used as the input to the subsequent model.

(13) $$F_{mfeb}=F_{tamp}+F_{msf}$$

### 3.4. Modulation Fusion Module

To effectively mitigate feature sparsity encountered during the decoding stages, we propose the MFM (Figure 1c). This module progressively integrates features extracted by the MFEB, while employing a dynamic weighting mechanism to adaptively emphasize and enhance critical feature representations. Specifically, the MFM module at the *i*-th stage receives feature inputs from two distinct sources: $F_{\mathrm{mfeb}}^{i}\in R^{\left(\frac{W}{2^{3-i}}\right)\times \left(\frac{H}{2^{3-i}}\right)\times \frac{C}{2^{i}}}$, extracted from the convolution operation after MFEB, and $F_{\mathrm{decoder}}^{i}\in R^{\left(\frac{W}{2^{3-i}}\right)\times \left(\frac{H}{2^{3-i}}\right)\times \frac{C}{2^{i}}}$, produced by the decoder at the corresponding stage. Here, *i* denotes the index of the current MFM module within the multi-stage fusion process. The fusion network comprises three such MFM modules. Both feature maps are first processed by a global pooling operation, yielding intermediate representations $P_{mfeb}^{A}\in R^{\frac{C}{2^{i}}\times 1}$ and $P_{decoder}^{A}\in R^{\frac{C}{2^{i}}\times 1}$. Subsequently, the features are merged via element-wise addition. The combined features are subsequently fed into an MLP for nonlinear transformation and dimensionality reduction, followed by channel-wise softmax normalization to generate adaptive fusion weights.

(14) $$y_{A}=\mathrm{MLP}\left(P_{a}^{A}+P_{b}^{A}\right)$$

(15) $$A^{i}=\mathrm{softmax}\left(y_{A}\right)$$

The values in $A^{i}$ represent the relative importance of $F_{\mathrm{mfeb}}^{i}$ and $F_{\mathrm{decoder}}^{i}$ within the synthetic network. These weights are applied to the input features through an element-wise multiplication, enabling feature modulation. This modulation process effectively highlights critical representations and helps preserve fine-grained structural details.

(16) $$F_{s}=A^{i}\cdot F_{\mathrm{mfeb}}^{i}+A^{i}\cdot F_{\mathrm{decoder}}^{i}$$

The modulated features $F_{s}$ are then concatenated with the original input $F_{\mathrm{decoder}}^{i}$, then passed through a convolutional layer to generate the final output feature map $F_{\mathrm{mfm}}^{i}$.

(17) $$F_{\mathrm{mfm}}^{i}=\mathrm{Conv}\left(\mathrm{Concat}\left(F_{s},F_{\mathrm{decoder}}^{i}\right)\right)$$

### 3.5. Implicit Neural Representation Block

**Figure 4:**
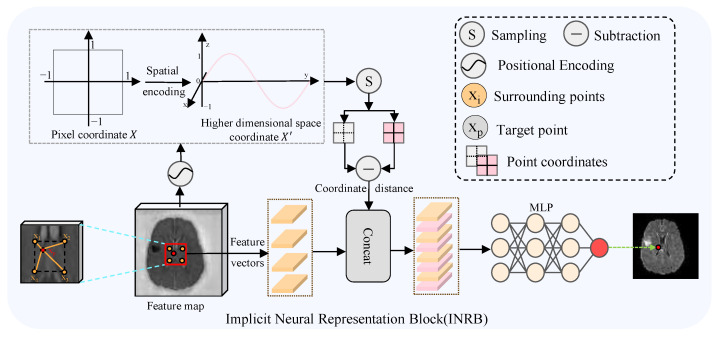
Architecture of the proposed Implicit Neural Representation Block (INRB), highlighting its two core components: pixel coordinate encoding and the multi-layer perceptron (MLP) decoder. The red frame marks a local feature region.

Figure 4 depicts the architecture of the INRB, which learns the implicit transformation from source modality features to the target image. Specifically, each predicted pixel $x_{p}$ is continuously described by the surrounding four locally related points $x_{i}$ (i=1,2,3,4). These pixel points are recorded in the relative coordinate set $X\in R^{H\times W\times 2}$, where the “2” denotes the horizontal and vertical coordinates. Additionally, we employ periodic spatial encoding to project these coordinates *X* are mapped into a higher-dimensional space $R^{2L}$ to enhance the recovery of high-frequency details. The encoding procedure can be expressed as:

(18) $$X^{\prime }=\psi \left(X\right)$$

(19) $$\psi \left(x\right)==[\sin\left(x\right),\cos\left(x\right),\ldots ,\sin\left(2^{L-1}x\right),\cos\left(2^{L-1}x\right)]$$

The function ψ(·) denotes the coordinate mapping mechanism. *x* represents the coordinate values of *X*, which are normalized to the range [−1,1]. The hyperparameter *L* determines the dimensionality of the encoding, and in our experiments, we set L=4. Then, based on nearest-neighbor interpolation, four local related points $x_{i}$ (i=1,2,3,4) are used as input features for the target point $x_{p}$. Simultaneously, by calculating the positional offsets between the target point and the local related points, we obtain the corresponding relative position encoding. Subsequently, we combine the local feature vectors $f_{x_{i}}\in R^{c}$ and their corresponding position encodings $P_{x_{i}}\in R^{2L}$ to form the intermediate features $F_{x_{i}}\in R^{c+2L}$.

(20) $$F_{x_{i}}=\mathrm{concat}\left(f_{x_{i}},P_{x_{i}}\right)$$

Finally, these are input into the decoder $f_{\theta }$, where the weighted average of predictions from neighboring grids is computed to achieve a continuous translation. This process can be regarded as implicit neural interpolation. This procedure can be formulated as:

(21) $$G\left(x_{p}\right)=\sum_{i\in \{1,2,3,4\}}w_{i}f_{\theta }\left(F_{x_{i}}\right)$$

(22) $$S=\sum_{i\in \{1,2,3,4\}}s_{i}$$

(23) $$w_{i}=\frac{s_{i}}{S}\;i=1,2,3,4$$

Here, $G(x_{p})$ represents the final pixel value at point $x_{p}$. Let $s_{i}$ denote the area of the region between the target point $x_{p}$ and a local related point $x_{i}$. $w_{i}$ denotes the local integration weight, which satisfies the condition that the sum of all $w_{i}$ equals 1. $f_{\theta }$ represents the MLP layer.

### 3.6. Loss Function

Here, we design the HMF-MambaINR as part of an adversarial framework, a conditional discriminator *D* operating on image patches is employed [40]. This discriminator is used to distinguish between the actual target images $y_{\mathrm{act}}$ and the synthetic target images $y_{\mathrm{syn}}$ generated by the generator *G*. Assuming that the training set contains pairs of source and target images for each example, we train the generator *G* using a composite objective function that accounts for both pixel-level reconstruction error and adversarial feedback:

(24) $$\begin{array}{ccc} L_{G} & \;\;=\lambda_{\mathrm{pix}}E\left[∥y_{\mathrm{syn}}-y_{\mathrm{act}}{∥}_{1}\right]-\; & \;\;\;\;\;\;\;\;\;\;\lambda_{\mathrm{adv}}\left\{E\left[D{\left(y_{\mathrm{act}}|X\right)}^{2}\right]+E\left[{\left(D(y_{\mathrm{syn}}|X)-1\right)}^{2}\right]\right\} \end{array}$$

Here, E represents the expectation, and $y_{\mathrm{syn}}=G\left(X\right)$ denotes the synthetic image generated by the generator *G* from the source image *X*. $\lambda_{\mathrm{pix}}$ and $\lambda_{\mathrm{adv}}$ are the weight coefficients of the pixel-by-pixel loss term and the adversarial loss term, which are 1 and 100 respectively. Meanwhile, the discriminator *D* is trained using adversarial loss to optimize its ability to to differentiate authentic target images from synthetically generated ones:

(25) $$L_{D}=E\left[D{\left(y_{\mathrm{act}}|X\right)}^{2}\right]+E\left[{\left(D(y_{\mathrm{syn}}|X)-1\right)}^{2}\right]$$

## 4. Experiments

### 4.1. Dataset

To demonstrate the effectiveness of our approach, experiments were conducted on two commonly used brain MRI datasets: the BraTS 2020 dataset [41,42,43] and the IXI dataset [44].

BraTS Dataset [41,42,43]. We used the BraTS 2020 dataset, which selected multi-parametric magnetic resonance imaging (mpMRI) scans collected from 240 patients with confirmed diffuse glioma. Four distinct MRI modalities constitute the dataset: T1-weighted (T1), T1c (contrast-enhanced T1), T2-weighted (T2), and FLAIR. In this study, we utilize the T1, T2, and FLAIR modalities to evaluate the effectiveness of the proposed method. Each modality volume has a spatial resolution of 240×240×155 voxels. For each subject, we extract 60 axial 2D slices containing brain tissue for subsequent analysis.Each axial slice was cropped to a 200×200 pixel image from the central region, then resized to 256×256 pixels. For the purposes of training and evaluating the model, we divided the data from the 240 subjects into training (70%), validation (10%), and testing (20%) sets. All images were linearly scaled to ensure their intensity values fell within the [0,1] range.

IXI Dataset [44]. The IXI dataset consists of brain images acquired using T1, T2, and PD weighting protocols, collected from healthy subjects using multiple scanners with varying magnetic field strengths. We selected a subset containing 6000 paired 2D slices across the three modalities. All slices were center-cropped and resized to 256×256 pixels, and intensities were normalized to [0,1]. The data distribution involves a random split, comprising a training set (70%), a validation set (10%), and a test set (20%).

### 4.2. Comparison Methods

To thoroughly assess the performance of the proposed HMF-MambaINR model, we selected representative methods from the current field of multi-modal medical image synthesis as baseline comparisons, including HiNet [24], PTNet [45], CACR-Net [25], ResVIT [10], INR-ECGAN [39] and IxI-Mamba [14]. These methods can be broadly summarized as follows:

HiNet [24] employs modality-specific subnetworks to learn representations and integrates a fusion network to derive a shared feature representation across different modalities, facilitating target image synthesis. PTNet [45] adopts a multi-scale pyramid Transformer, emphasizing cross-scale context modeling to enhance image synthesis performance. CACR-Net [25] proposes a confidence-guided fusion and cross-modal refinement network, further refining the target modality image. ResVIT [10] employs a hybrid CNN-Transformer framework, combining residual Transformer blocks and a channel compression module for multi-modal medical image synthesis. INR-ECGAN [39] is the first to explore implicit neural representations for modeling a continuous mapping from SAR features to optical images. IxI-Mamba [14] is based on selective state space modeling, effectively capturing long-range contextual dependencies while preserving local structural accuracy, balancing global consistency and detail representation.

### 4.3. Experimental Results

We conducted three multi-modal image synthesis experiments using the BraTS 2020 dataset: T2 was synthesized from T1 and FLAIR (T1,FLAIR→T2), T1 from T2 and FLAIR (T2,FLAIR→T1), and synthesizing FLAIR from T1 and T2 (T1,T2→FLAIR). For each synthesis task, we presente’d quantitative analyses alongside qualitative illustrations in the following sections.

**Figure 5:**
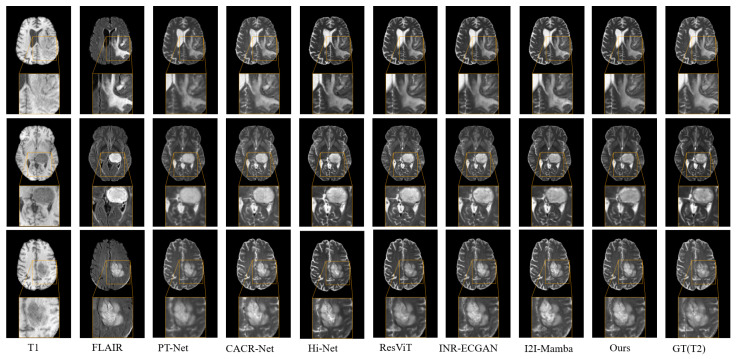
Visual comparison of the proposed HMF-MambaINR with other methods on the synthetic T2 task (T1, FLAIR → T2) using the BraTS 2020 dataset.

Using the T2 synthesis task as a representative example, we presented both quantitative evaluations and visual comparison results of different methods in Figure 5 and Table 1, respectively. Compared to the I2I-Mamba, our proposed method achieved improvements of 0.47 in PSNR, 0.014 in SSIM, and 0.015 in NMSE. Furthermore, as illustrated in Figure 5, HMF-MambaINR not only preserved the structural integrity of the synthesized T2 images but also produced more accurate pixel distributions and sharper visual details, clearly outperforming other methods in both perceptual quality and structural fidelity.

**Figure 6:**
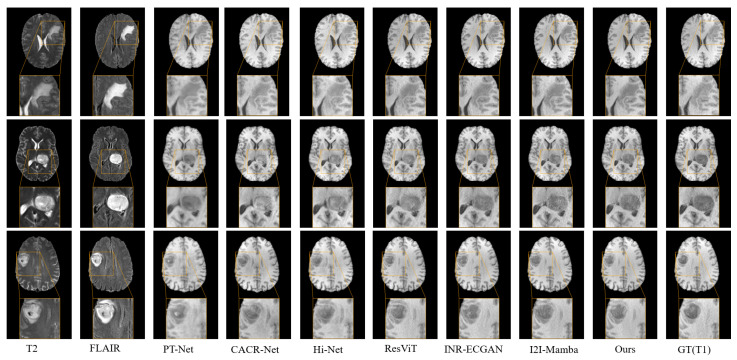
Visual comparison of the proposed HMF-MambaINR with other methods on the synthetic T1 task (T2, FLAIR → T1) using the BraTS 2020 dataset.

For the T1 synthesis task, Table 1 presents the quantitative results for this task. Our HMF-MambaINR model markedly surpassed existing methods across key metrics, including PSNR, SSIM, and NMSE. These results demonstrated that our model effectively integrated information from different stages and scales, thereby enhancing synthesis performance. Figure 6 provided visual comparisons of T1 images synthesized by our method and by representative baselines. The T1 modality images synthesized by the HMF-MambaINR model exhibited higher quality than those synthesized by the other methods. These improvements included clearer tissue delineation and fewer artifacts, producing visually superior high-quality outputs.

**Figure 7:**
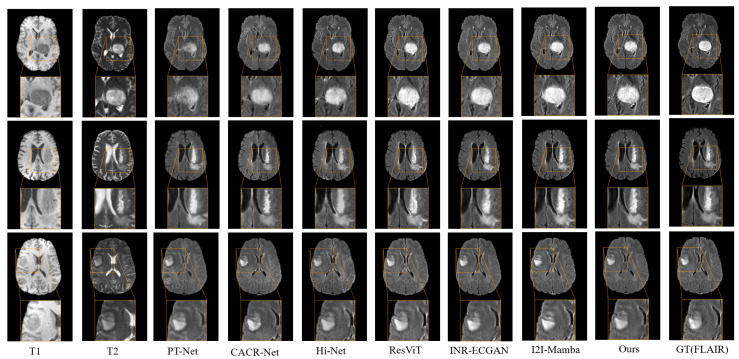
Visual comparison of the proposed HMF-MambaINR with other methods on the synthetic FLAIR task (T1, T2 → FLAIR) using the BraTS 2020 dataset.

In the final T1, T2 → FLAIR synthesis task, we presented quantitative comparisons with baseline methods in Table 1 and provided qualitative visualizations in Figure 7 to illustrate the perceptual fidelity of the synthesized results. Clearly, CACR-Net and Hi-Net performed poorly in this task, whereas the proposed method demonstrated substantial gains across all three evaluation metrics. Furthermore, compared to I2I-Mamba, HMF-MambaINR demonstrated improvements of 0.51, 0.013, and 0.008 in PSNR, SSIM, and NMSE, respectively. In the yellow box region of Figure 7, the FLAIR modality images synthesized using our method exhibited more precise pixel details, further confirming the advantages of our approach in cross-modality MRI synthesis.

In addition, we conducted complementary experiments on the IXI dataset to further evaluated the generalizability of our method on healthy brain MRI data. Specifically, we designed three synthesis tasks: synthesizing T2 using T1 and PD as inputs (T1,PD→T2), synthesizing PD using T1 and T2 as inputs (T1,T2→PD), and synthesizing T1 using T2 and PD as inputs (T2,PD→T1). These tasks covered diverse modality combinations, offering a thorough evaluation of the model’s performance across both pathological and non-pathological conditions.

**Figure 8:**
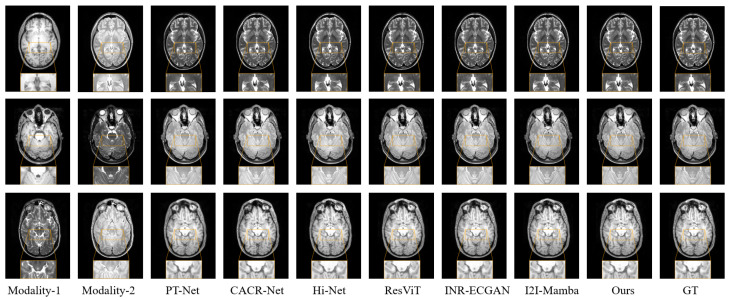
Visual Comparison of Proposed HMF-MambaINR Results on the IXI Dataset. T1, PD → T2 (first row), T1, T2 → PD (second row), and T2, PD → T1 (third row).

Table 2 presented the PSNR, SSIM, and NMSE scores for the multi-input single-output synthesis tasks. Our proposed MFE-MambaINR model consistently achieved the highest performance across all tasks. It outperformed convolution-based models, Transformer-based architectures, and the original Mamba-based baseline. Averaged across the three benchmark tasks, our method consistently outperformed INR-ECGAN, delivering a PSNR improvement of 0.89, a 0.010 increase in SSIM, and a 0.02 reduction in NMSE. Furthermore, it surpassed ResViT, with gains of 0.43 in PSNR, 0.050 in SSIM, and a 0.002 decrease in NMSE. Compared to I2I-Mamba, our approach achieved comparable improvements—0.41 higher PSNR, 0.006 higher SSIM, and a 0.002 lower NMSE. Figure 8 illustrated the qualitative comparison of synthesized results for three tasks: T1, PD → T2 (first row), T1, T2 → PD (second row), and T2, PD → T1 (third row). As shown, MFE-MambaINR generated images with fewer artifacts and clearer tissue structures compared to convolution-, Transformer-, and Mamba-based models, further demonstrating its effectiveness in high-fidelity modality synthesis.

### 4.4. Model Complexity

We assessed the model complexity of our approach alongside several state-of-the-art methods by measuring FLOPs, parameter counts, and inference times, as summarized in Table 3. Although MFE-MambaINR incurred slightly higher FLOPs and parameters than I2I-Mamba, it improved image synthesis performance, especially in preserving fine details and structural fidelity. This gain underscored the efficacy of integrating Multi-Scale Feature Enhancement and INR modules to boost performance while maintaining controlled complexity. In terms of inference efficiency, MFE-MambaINR requires slightly longer inference time compared to I2I-Mamba, but the increase is modest and acceptable given the notable improvements in synthesis quality. In contrast to ResViT, MFE-MambaINR achieved better synthesis quality with fewer parameters and reduced FLOPs. These results demonstrated that our model struck a favorable balance between synthesis accuracy and computational efficiency, enhancing performance while minimizing resource demands. This rendered our model better suited for practical medical image processing tasks in clinical settings.

### 4.5. Radiology Evaluation and Error Analysis

To more comprehensively assess the quality of the synthesized images produced by the proposed method, we designed and conducted a subjective evaluation experiment involving three experienced radiologists. The experts independently evaluated the generated images using multiple criteria. The images were anonymized and arranged randomly, and the radiologists were blinded to the source of each image to prevent potential bias throughout the evaluation. This study evaluated three tasks on the BraTS dataset, with 12 images per task, for a total of 36 images.

The evaluation focused on three key assessment criteria: overall visual fidelity, contrast characteristics, and structural edge clarity. A five-level Likert scale was employed to assess both overall visual fidelity and contrast characteristics, with ratings defined as: 1 for “unacceptable,” 2 for “poor,” 3 for “acceptable,” 4 for “good,” and 5 for “excellent”. Structural edge clarity was assessed using a three-point scale, where 1 indicated “unclear edges”, 2 indicated “discernible edges”, and 3 indicated “clear edges”. As shown in Table 4, our model achieved scores of 4.75, 4.53, and 2.65 in image quality, image contrast, and structural contour, respectively, significantly outperforming other methods. These results indicated that our approach can more accurately capture fine anatomical structures and demonstrated higher clinical value.

**Figure 9:**
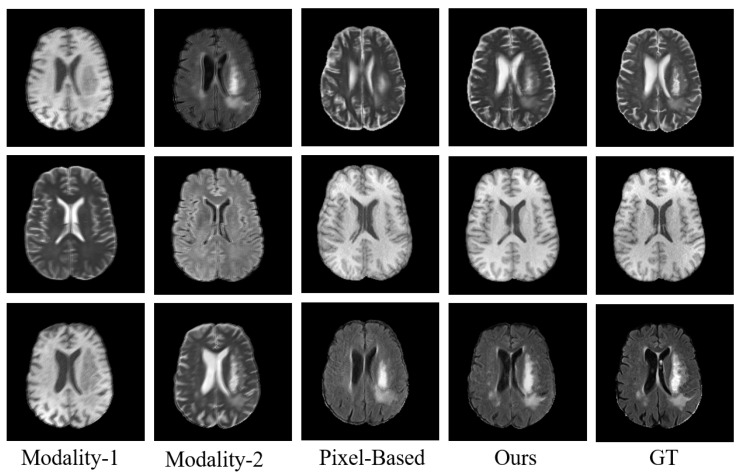
Visual Comparison of Proposed HMF-MambaINR and Pixel-Based (INR replaced by a pixel-based decoder) Results on the BraTS 2020 Dataset (motion artifacts). T1, Flair → T2 (first row), T2, Flair → T1 (second row), and T1, T2 → Flair (third row).

To further evaluate the robustness of our model, we conducted additional experiments on the BraTS 2020 dataset by introducing simulated motion artifacts, aiming to mimic realistic and imperfect clinical acquisition conditions. Under motion corruption, the source modality was deliberately subjected to spatially varying motion-induced ghosting and blurring effects, which are commonly observed in routine MRI scans due to patient movement. The proposed model was then evaluated without any additional fine-tuning to assess its generalization capability under such challenging conditions. As shown in Table 5, we compared the performance of our model under normal conditions and in the presence of motion artifacts. The results indicated that our model exhibited only a limited performance drop when motion corruption was introduced. As illustrated in Figure 9, we visualized the outputs of our model on different MRI modalities. The results demonstrated that, despite some degradation caused by motion artifacts, our model successfully recovered fine structural details and maintained contrast consistent with the ground truth. To further investigate the contribution of the INR module, we replaced it with a parameter-matched Pixel-Based decoder and generated the corresponding outputs. We observed that the Pixel-Based decoder produced noticeable blurring, loss of structural details, and visible boundary ghosting, indicating that the INR-based model better preserved anatomical fidelity and fine-grained features.

### 4.6. Ablation Study

**Figure 10:**
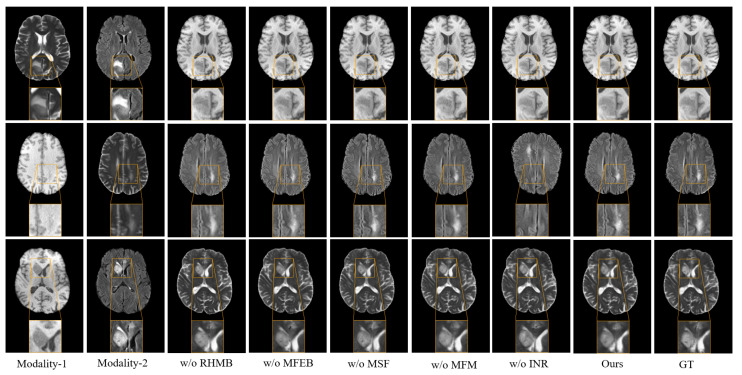
Visualization of ablation experiment outcomes using the BraTS 2020 dataset.

To verify the contribution of each constituent part of the proposed network, comprehensive ablation studies were performed on the BraTS 2020 dataset. Qualitative and quantitative assessments of the experimental data are detailed in Figure 10 and Table 6.

1. The Role of the RHMB Module. To assess the effectiveness of the RHMB module in extracting deep semantic features and local texture information from images, as well as its contribution to enhancing the quality of synthesized images for missing modalities, ablation experiments were designed. Specifically, a variant model, named w/o RHMB, was created by replacing the RHMB module with a convolutional layer of comparable parameter count. This model performed only basic convolution operations on the features, lacking the context-enhancing mechanism. The experimental results indicated a significant degradation in the structural integrity and detail representation of the synthesized images when the RHMB module was removed, demonstrating the importance and effectiveness of RHMB in multi-modal medical image synthesis tasks.
2. The Impact of the MFEB Module. To assess the contribution of the MFEB to the overall model performance, we performed an ablation analysis by retaining only the connection and convolution operations in the MFEB module, referred to as w/o MFEB. The experiment was designed to quantify the effect of multi-stage and multi-scale feature extraction on the quality of modality synthesis. As reported in the results Table 6, eliminating the MFEB module led to a significant performance drop. This finding highlighted the critical importance of jointly leveraging complementary features extracted across different stages and scales—particularly those closely aligned with the target modality—in improving the fidelity and perceptual quality of the synthesized images.
3. The Impact of the MSF Module. To validate the performance impact of the multi-scale feature fusion module in the MFEB module, relevant ablation experiments were designed. Specifically, a baseline model was constructed without the MSF module, named w/o MSF, and was replaced with standard convolution operations for comparison. Experimental evidence supported the conclusion that after removing the MSF module, the model performed poorly in the multimodal feature fusion task, highlighting the key role of this module in improving the quality of image synthesis.
4. The Impact of the MFM Module. In order to better study the effectiveness of the MFM module in enhancing the feature representation capability of the network, we constructed a variant model, named w/o MFM, in which the MFM module was replaced by a feature addition module. As shown in Table 6 and Figure 10, the MFM module was able to effectively alleviate the feature sparsity problem in the encoding process and significantly enhanced the overall performance of the model.
5. The Importance of the INR Module. To explore the contribution of INRB to image synthesis tasks, corresponding ablation experiments were conducted. Specifically, the INR module in the original model was replaced with a parameter-matched standard MLP layer without coordinate embedding to form a comparison model, named w/o INR. This experiment was designed to analyze the specific contributions of continuous implicit representations in preserving image details, structural restoration, and texture reconstruction. The experimental results confirmed that the INR decoder significantly enhanced the continuity and detail retention of the synthesized images, thereby verifying the effectiveness and potential of modeling medical images using continuous functions.

## 5. Discussion

This paper introduced a hierarchical multi-scale feature fusion network designed for multi-modal MRI synthesis. Existing CNN-based methods struggled to effectively model non-local dependencies between distant anatomical structures due to their limited receptive field, which restricted their performance in global structural modeling. While Transformer-based methods mitigated this issue to some extent by employing self-attention operators, they introduced quadratic computational complexity with respect to sequence length, thereby limiting inference efficiency. In contrast to these approaches, the proposed HMF-MambaINR model integrated the context-aware modeling capability of state space models (SSM) with the local precision of convolutional neural networks (CNN), enabling efficient modeling of long-range dependencies in medical images without increasing computational burden. Additionally, by incorporating a multi-stage, multi-scale feature extraction and fusion mechanism, the proposed method facilitated cross-layer information exchange by integrating multi-level features from different stages and scales. This design allowed the simultaneous aggregation of fine-grained texture details and high-level semantic representations, thereby enhancing both the richness and robustness of feature representations. Unlike traditional explicit decoders, an INR decoder was designed in the decoding stage. This decoder took image coordinates as input and performed continuous mapping of the fused features through a multi-layer MLP. Owing to its ability to model spatial continuity, this module demonstrated superior expressive power when handling complex boundary structures in medical images. From a clinical deployment perspective, the proposed HMF-MambaINR framework demonstrated favorable feasibility for real-world applications. The use of Mamba-based state space modeling enabled efficient long-range dependency modeling with linear computational complexity, which was well suited for high-resolution MRI data in routine clinical environments. Moreover, the proposed method operated on standard MRI modalities without requiring additional annotations or specialized acquisition protocols, facilitating seamless integration into existing clinical workflows. The implicit decoder further supported continuous and structurally consistent image synthesis, enhancing robustness across heterogeneous scanners and imaging settings.

## 6. Conclusions

In this paper, we proposed a novel learning-based model for cross-modality MRI synthesis. The proposed model, named HMF-MambaINR, employed residual mixed Mamba blocks to model both spatial and channel context, effectively capturing contextual information in medical imaging while avoiding the quadratic complexity inherent in self-attention mechanisms. To further enhance feature representation, we introduced a multi-feature, multi-scale feature extraction module (MFEB), which extended the network’s width while maintaining its depth. This design helped the model better extract shallow texture details and global semantic features. In addition, we developed a modulation fusion module (MFM) to dynamically integrate multi-scale features and effectively alleviate feature sparsity during the decoding stage. Finally, we designed an implicit decoder that continuously mapped the fused features back to the medical image, enabling more effective preservation of fine textures and structural details. Compared with current mainstream methods—including convolutional, Transformer-based, and Mamba-based models—our approach delivered consistently improved performance across all evaluated tasks.

## 7. Limitations and Future Work

Despite these promising properties, several limitations remain and should be addressed in future work. First, the current evaluation is primarily conducted on public benchmark datasets under controlled experimental settings, which may not fully capture the diversity and complexity of raw clinical data encountered in real-world practice. As a result, the generalization performance under diverse acquisition protocols, scanner vendors, and patient populations requires further investigation. Second, although image quality metrics and expert-based subjective evaluations indicate higher synthesis quality, these assessments do not directly reflect the clinical utility of the synthesized images in downstream tasks such as segmentation, registration, or diagnostic decision-making. Future research will focus on closing the gap between methodological validation and real-world clinical deployment. In particular, the proposed framework will be evaluated on real-world clinical datasets obtained under routine clinical settings to comprehensively examine robustness and generalizability in practical scenarios. In addition, task-driven evaluations will be conducted by incorporating synthesized images into downstream clinical pipelines to quantitatively measure their impact on clinically relevant tasks. Beyond evaluation, integrating uncertainty-aware modeling or diffusion-based probabilistic mechanisms [46] into the HMF-MambaINR framework could further enhance its ability to represent complex anatomical structures, blurred boundaries, and uncertain scenarios, thereby improving reliability in real-world clinical deployment.

## Figures and Tables

**Table 1:** Performance on different MRI modality translations on the BraTS 2020 dataset (↑ indicates higher is better; ↓ indicates lower is better; bold denotes the best result).

Method	$T_{1}$, FLAIR → $T_{2}$			T2, FLAIR → T1			T1, T2 → FLAIR		
	**PSNR **↑	**SSIM **↑	**NMSE **↓	**PSNR **↑	**SSIM **↑	**NMSE **↓	**PSNR **↑	**SSIM **↑	**NMSE **↓
PT-Net	25.68±1.649	0.883±0.028	0.077±0.026	25.31±1.611	0.871±0.026	0.029±0.014	23.12±2.697	0.806±0.037	0.098±0.061
CACR-Net	24.64±1.317	0.882±0.025	0.064±0.028	25.23±2.025	0.870±0.028	0.030±0.020	21.52±2.375	0.798±0.032	0.106±0.078
Hi-Net	24.75±1.407	0.880±0.027	0.062±0.025	25.45±1.960	0.866±0.026	0.028±0.019	21.97±1.988	0.809±0.032	0.096±0.073
ResViT	26.32±1.874	0.889±0.025	0.067±0.027	25.86±1.690	0.871±0.027	0.029±0.012	23.79±2.691	0.811±0.038	0.081±0.048
INR-ECGAN	26.29±1.766	0.890±0.032	0.064±0.030	25.77±1.812	0.872±0.030	0.027±0.022	23.73±2.510	0.810±0.033	0.084±0.053
I2I-Mamba	26.36±1.934	0.887±0.030	0.067±0.024	25.89±1.764	0.869±0.027	0.026±0.014	23.83±2.650	0.805±0.035	0.078±0.043
**Ours**	26.83±1.660	0.901±0.032	0.052±0.029	26.33±1.811	0.881±0.028	0.022±0.013	24.34±2.509	0.818±0.036	0.070±0.041

**Table 2:** Performance on different MRI modality translations on the IXI Dataset.(↑ indicates higher is better; ↓ indicates lower is better; bold denotes the best result).

Method	$T_{1}$, PD → $T_{2}$			$T_{1}$, $T_{2}$ → PD			$T_{2}$, PD → $T_{1}$		
	**PSNR ↑**	**SSIM ↑**	**NMSE ↑**	**PSNR ↑**	**SSIM ↑**	**NMSE ↓**	**PSNR ↑**	**SSIM**	**NMSE ↑**
PT-Net	28.14 ± 1.758	0.927 ± 0.016	0.032 ± 0.012	28.88 ± 1.919	0.934 ± 0.016	0.013 ± 0.008	27.33 ± 2.026	0.883 ± 0.032	0.029 ± 0.014
CACR-Net	28.04 ± 1.745	0.926 ± 0.015	0.028 ± 0.011	29.18 ± 1.966	0.939 ± 0.014	0.013 ± 0.007	27.83 ± 1.918	0.882 ± 0.033	0.029 ± 0.015
Hi-Net	27.76 ± 1.727	0.928 ± 0.014	0.036 ± 0.014	28.41 ± 1.784	0.940 ± 0.013	0.016 ± 0.009	27.45 ± 1.785	0.879 ± 0.033	0.030 ± 0.014
ResViT	29.35 ± 1.634	0.930 ± 0.014	0.025 ± 0.008	29.85 ± 1.736	0.940 ± 0.013	0.013 ± 0.005	27.77 ± 2.113	0.890 ± 0.025	0.026 ± 0.013
INR-ECGAN	28.39 ± 1.700	0.927 ± 0.014	0.026 ± 0.011	29.41 ± 1.750	0.937 ± 0.013	0.012 ± 0.007	27.71 ± 2.104	0.882 ± 0.030	0.027 ± 0.017
I2I-Mamba	29.25 ± 1.580	0.928 ± 0.014	0.027 ± 0.010	29.79 ± 1.823	0.936 ± 0.014	0.012 ± 0.007	27.98 ± 2.010	0.892 ± 0.027	0.026 ± 0.012
**Ours**	**29.62 ± 1.625**	**0.934 ± 0.015**	**0.023 ± 0.008**	**30.10 ± 1.868**	**0.943 ± 0.014**	**0.010 ± 0.006**	**28.47 ± 2.056**	**0.898 ± 0.023**	**0.025 ± 0.013**

**Table 3:** Model complexity comparison with state-of-the-art approaches.

	PT-Net	CACR-Net	Hi-Net	ResViT	INR-ECGAN	I2I-Mamba	Ours
FLOPs (G)	19.28	12.44	8.33	139.12	111.23	89.58	105.84
Params (M)	28.14	6.79	4.26	123.4	96.63	103.8	107.21
Inference times (ms)	52.23	45.57	49.35	58.49	127.83	56.54	220.21

**Table 4:** Subjective evaluation of visual quality for different methods on the BraTS 2020 Dataset.

	Ratings (Mean ± Standard Deviation)						
	**PT-Net**	**CACR-Net**	**Hi-Net**	**ResViT**	**INR-ECGAN**	**I2I-Mamba**	**Ours**
Image Quality	4.22 ± 0.12	3.86 ± 0.14	3.94 ± 0.06	4.38 ± 0.11	4.36 ± 0.12	4.45 ± 0.12	4.75 ± 0.10
Image Contrast	4.11 ± 0.07	3.72 ± 0.16	3.74 ± 0.15	4.25 ± 0.07	4.20 ± 0.08	4.21 ± 0.07	4.53 ± 0.06
Structural Contours	2.37 ± 0.09	2.18 ± 0.17	2.24 ± 0.11	2.41 ± 0.06	2.43 ± 0.08	2.52 ± 0.07	2.65 ± 0.04

**Table 5:** Performance comparison under normal and motion-artifact conditions. (↑ indicates higher is better; ↓ indicates lower is better).

Dataset	PSNR ↑	SSIM ↑	NMSE ↓
T1, Flair → T2			
Motion artifacts	22.49±1.656	0.784±0.049	0.103±0.057
Normal	26.83±1.660	0.901±0.032	0.052±0.029
T2, Flair → T1			
Motion artifacts	22.65±1.439	0.781±0.046	0.052±0.031
Normal	26.33±1.811	0.881±0.028	0.022±0.013
T1, T2 → Flair			
Motion artifacts	21.93±2.103	0.719±0.051	0.129±0.806
Normal	24.34±2.509	0.818±0.036	0.070±0.041

**Table 6:** Ablation study results on different MRI modality translation tasks on the BraTS2020 dataset.

Method	$T_{1}$, Flair → $T_{2}$			$T_{2}$, Flair → $T_{1}$			$T_{1}$, $T_{2}$ → Flair		
	**PSNR **↑	**SSIM **↑	**NMSE **↓	**PSNR **↑	**SSIM **↑	**NMSE **↓	**PSNR **↑	**SSIM **↑	**NMSE **↓
w/o RHMB	26.34 ± 1.718	0.883 ± 0.035	0.068 ± 0.035	25.82 ± 1.724	0.867 ± 0.034	0.033 ± 0.017	23.87 ± 2.512	0.801 ± 0.035	0.082 ± 0.051
w/o MFEB	26.50 ± 1.873	0.886 ± 0.029	0.061 ± 0.027	26.02 ± 1.814	0.871 ± 0.030	0.028 ± 0.014	24.01 ± 2.544	0.809 ± 0.043	0.079 ± 0.044
w/o MSF	26.62 ± 1.951	0.890 ± 0.031	0.058 ± 0.026	26.11 ± 1.807	0.874 ± 0.028	0.025 ± 0.013	24.13 ± 2.514	0.813 ± 0.037	0.077 ± 0.041
w/o MFM	26.70 ± 1.946	0.892 ± 0.030	0.059 ± 0.029	26.15 ± 1.839	0.873 ± 0.027	0.027 ± 0.013	24.18 ± 2.730	0.811 ± 0.038	0.075 ± 0.042
w/o INR	26.46 ± 1.731	0.889 ± 0.029	0.065 ± 0.023	26.13 ± 1.844	0.877 ± 0.026	0.024 ± 0.014	24.18 ± 2.597	0.815 ± 0.037	0.081 ± 0.060
**Ours**	**26.83 ± 1.660**	**0.901 ± 0.032**	**0.052 ± 0.029**	**26.33 ± 1.811**	**0.881 ± 0.028**	**0.022 ± 0.013**	**24.34 ± 2.509**	**0.818 ± 0.036**	**0.070 ± 0.041**

## Data Availability

Publicly available datasets were analyzed in this study. These data can be found here: the BraTS 2020 dataset (https://www.med.upenn.edu/cbica/brats2020/, accessed on 22 November 2023), and the IXI dataset (https://brain-development.org/ixi-dataset/, accessed on 16 May 2025).
